# Towards the practical realization of high-performance Ag_2_Se-based thermoelectric coolers

**DOI:** 10.1080/14686996.2026.2641882

**Published:** 2026-03-12

**Authors:** Feng Jiang, Zhengtao Wang, Wen Zhong, Yifan Zhou, Zhengyang Zhou, Longzhi Wu, Jiang Chen, Yao Xu, Xiaodong Wang, Feng Cao, Qian Zhang, Jun Mao

**Affiliations:** a School of Materials Science and Engineering, and Institute of Materials Genome & Big Data, Harbin Institute of Technology (Shenzhen), Shenzhen, P.R. China; b Institute of Special Environments Physical Sciences, Harbin Institute of Technology (Shenzhen), Shenzhen, P.R. China; c School of Science, Harbin Institute of Technology (Shenzhen), Shenzhen, P.R. China; d State Key Laboratory of Precision Welding & Joining of Materials and Structures, Harbin Institute of Technology, Harbin, P.R. China; e School of Materials Science and Engineering, Shenzhen Key Laboratory of New Materials Technology, Shenzhen, P.R. China

**Keywords:** Thermoelectric cooling, Ag_2_Se, compositional homogeneity

## Abstract

Ag_2_Se-based materials with promising room-temperature thermoelectric performance and excellent mechanical properties have been known for decades. However, the fabrication of the Ag_2_Se-based devices toward the practical application of electronic cooling has seldom been reported. Herein, the synthesis of Ag_2_Se material with a diameter of 25.4 mm was achieved. The homogeneous elemental distribution and similar thermoelectric properties demonstrate the high uniformity of the Ag_2_Se sample. In addition, ~90 Ag/Ag_2_Se/Ag legs can be obtained from a single Ag_2_Se plate, and the interfacial contact resistivity of the Ag/Ag_2_Se varies from 0.8 to 3.1 μΩ cm^2^. Four thermoelectric devices based on n-type Ag_2_Se and p-type (Bi, Sb)_2_Te_3_ have been fabricated, and they achieve a cooling temperature difference of ~56 K at the hot-side temperature of 300 K, demonstrating the great potential of Ag_2_Se material for cooling applications.

## IMPACT STATEMENT

We synthesized Ag_2_Se samples with homogenous composition and thermoelectric properties, and successfully fabricated high-performance Ag_2_Se-based thermoelectric coolers with superior operational stability.

## GRAPHICAL ABSTRACT

**Figure:**
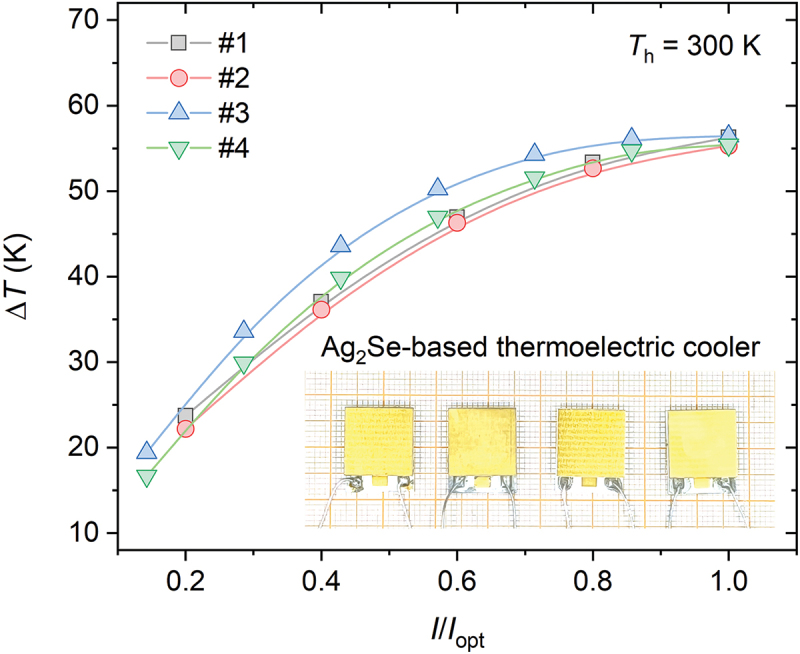

## 1. Introduction

Thermoelectric materials enabling the direct conversion between electricity and heat are crucial for heat harvesting and electronic refrigeration [1–3]. The performance of a thermoelectric device is strongly dependent on the properties of the materials, which is expressed by the dimensionless figure of merit, *zT* = *S*^2^*T*/*ρκ*, where *S*, *ρ*, *T*, and *κ* are the Seebeck coefficient, electrical resistivity, absolute temperature, and thermal conductivity, respectively [4–7]. Pursuing high *zT* values is essential for achieving a device with excellent performance.

For solid-state cooling applications, materials with high room-temperature *zT* values will be favored [8–11]. For example, Bi_2_Te_3_-based alloys have long been employed for commercial thermoelectric cooling applications due to their excellent performance near room temperatures [12–14]. However, the low natural abundance of the Te element and the poor mechanical properties of Bi_2_Te_3_-based materials have motivated the development of novel materials with high thermoelectric performance and superior mechanical properties.

Recently, Mg_3_Sb_2-*x*_Bi_*x*_-based materials have been developed and exhibit superior performance near room temperature [15–21] and excellent mechanical properties [22–26]. Moreover, thermoelectric devices with excellent cooling performance have been reported [27–33]. For example, a two-stage Mg_3_Bi_2_-based device was developed, and achieved a cooling temperature difference of 106.7 K at the hot-side temperature of 350 K [34]. In addition, a miniaturized Mg_3_Bi_2_-based cooler was also fabricated, a high cooling power density of 5.7 W cm^−2^ and a cooling speed of 65 K s^−1^ were achieved [35].

As another potential candidate, n-type Ag_2_Se has long been known as a high-performance thermoelectric material with a *zT* value of ~0.9 at room temperature [36]. Recently, Ag_2_Se-based thermoelectric devices have been successfully fabricated and have achieved high cooling performance [37–39]. To enable the practical application of Ag_2_Se in electronic cooling, it is critical to ensure the homogeneity of Ag_2_Se-based materials for the practical fabrication of Ag_2_Se-based thermoelectric coolers. However, such studies have seldom been reported.

Herein, the fabrication of the Ag_2_Se-based thermoelectric coolers has been realized. Ag_2_Se materials were prepared through ball-milling and sintering. In addition, a total of ~ 90 Ag/Ag_2_Se/Ag legs can be obtained from one Ag/Ag_2_Se/Ag plate with a diameter of 25.4 mm. The as-prepared samples not only exhibit good homogeneity but also present promising thermoelectric performance. In addition, four thermoelectric cooler devices based on the n-type Ag_2_Se and p-type (Bi, Sb)_2_Te_3_ have been fabricated, and they show a maximum temperature difference of ~ 56 K and a cooling power density of 1.56 W cm^−2^ at the hot-side temperature of 300 K.

## 2. Experimental method

### 2.1. Sample synthesis

Ag_2_Se samples were prepared by the mechanical alloying method. Silver (Ag rods, with a diameter of ~1.9 mm and a length of ~5.1 mm, ZNXC, 99.995%) and selenium (Se shots, with a diameter of ~2.1 mm, ZNXC, 99.999%) were weighed in total 50 g according to the nominal composition of Ag_2_Se_1.01_ and loaded into a stainless jar with two stainless balls (with a diameter of ~12.6 mm and a mass of 15.9 g) in a high vacuum glove box (with both oxygen and water level below 1 ppm). The mixed raw materials were ball-milled in a high-energy ball milling machine (SPEX 8000M, SPEX SamplePrep, LLC, USA) for 8 h (Once every 4 hours, with a 15-minute interval). The powders were then loaded in the graphite dies with a diameter of 10 mm, 12.7 mm, 20.0 mm, and 25.4 mm for densification at 523 K for 5 min using a homemade spark plasma sintering(SPS), under a pressure of 40 MPa (with a vacuum level of ~40 ppm).

### 2.2. Composition and microstructure characterization

The phase composition of the Ag_2_Se samples was examined by X-ray diffraction (XRD) with Cu *K*_α_ radiation (D/Max-2500 PC, Rigaku, Japan). The microstructure and surface morphology of the Ag_2_Se sample, as well as the interfacial morphology of the Ag_2_Se legs, were investigated by the scanning electron microscope (SEM, Crossbeam 350, ZEISS, Germany) equipped with energy dispersive X-ray spectroscopy (EDS). The chemical composition of the Ag_2_Se sample with a diameter of 25.4 mm was evaluated by the electron probe microanalysis (EMPA, JXA-8100, JOEL, Japan) with a voltage of 20.0 kV.

### 2.3. Thermoelectric properties characterization

The Ag_2_Se samples with different diameters were cut into bar-shaped samples with dimensions of about 2 mm × 2 mm × 10 mm for simultaneously measuring the electrical resistivity and Seebeck coefficient from 300 to 380 K on a commercial apparatus (CTA-3 Cryoall, China) under a helium atmosphere. The Ag_2_Se samples were cut into disks with dimensions of about 6 mm × 6 mm × 1 mm for the measurement of thermal diffusivity (LFA 457, Netzsch, Germany) from 300 to 380 K. The specific heat capacity of Ag_2_Se was measured by a differential scanning calorimeter (DSC 404F3, Netzsch, Germany) with an uncertainty of 3%, and the results are shown in Figure S1 (Supplementary Information). The density of the sample was measured by the Archimedean method. Eventually, the thermal conductivity is calculated by the product of thermal diffusivity, specific heat capacity, and sample density. The temperature-dependent Hall coefficients (*R*_H_) were measured using the van der Pauw method and a four-probe configuration under a reversible magnetic field of 1.5 T and an electrical current of 150 mA from 300 to 380 K. The Hall carrier concentration (*n*_H_) and Hall mobility (*μ*_H_) were calculated based on the following relationships *n*_H_ = 1/(e*R*_H_) and *μ*_H_ = *R*_H_/*ρ*, respectively.

### 2.4. Thermoelectric cooler fabrication and performance characterization

Silver was used as the contact layer for Ag_2_Se-based thermoelectric coolers. The Ag_2_Se plate was loaded into a graphite die with a diameter of 25.4 mm, with 1.0 g Ag powder placed on each side within the glove box. The Ag/Ag_2_Se/Ag joint was prepared by using the spark plasma sintering at 523 K under a pressure of 40 MPa for 5 min. Importantly, an additional pressure of 10 MPa was maintained during the cooling process to ensure good contact property [38]. The commercial p-type (Bi, Sb)_2_Te_3_ (RusTec LLC) was electroplated with Ni and applied as the p-type thermoelectric material. The thermoelectric cooler, consisting of 7 pairs of n-type Ag_2_Se and p-type (Bi, Sb)_2_Te_3_ legs, was assembled with the ceramic substrate that has been precoated with Cu and Ni layers, and with SnBi solder (Sn_42_Bi_58_, melting point 411 K). Then the assembly was subjected to reflow soldering at 458 K for 50 s with an external load. The contact electrical resistivity of the joints was measured using a homemade apparatus with alternating electrical currents [40].

The thermoelectric cooling performance of the Ag_2_Se-based device was measured by a homemade apparatus in a vacuum [41]. The cooling power (*Q*_c_) was evaluated by a reference sample with known thermal conductivity by using four thermocouples based on the one-dimensional Fourier heat conduction law.

(1) $$Q_{c}=-\mathrm{\kappa A}\frac{\mathrm{dT}}{\mathrm{dz}}$$

where *κ* and *A* are the thermal conductivity and the cross-sectional area of the reference sample, respectively. The cooling coefficient of performance (COP) is defined as the ratio of the *Q*_c_ and the input electrical power (*P*).

(2) $$\mathrm{COP}=Q_{c}/P$$

The temperature differences (Δ*T*) across the thermoelectric cooler under various currents were obtained under a steady condition that the hot-side temperature varies within 0.15 K in 60 s by subtracting the cold-side temperature (*T*_c_) from the hot-side temperature (*T*_h_), both measured with attached thermocouples [41]. The operational stability of the Ag_2_Se-based thermoelectric coolers is evaluated by cycling tests with alternating currents between 1 A and 6 A. At each current, the test duration was set to 40 s, and the cooling temperature differences at 1 A and 6 A were recorded.

### 2.5. Simulation of the cooling performance of the Ag_2_Se-based cooler

The cooling performance of the Ag_2_Se-based thermoelectric cooler was simulated by finite element analysis software (COMSOL Multiphysics) based on the thermoelectric properties of the n-type Ag_2_Se and the p-type (Bi, Sb)_2_Te_3_. The simulation parameters are presented in Table S1, Supplementary Information. The thickness of the contact layer for Ag_2_Se and (Bi, Sb)_2_Te_3_ is set as 200 μm and 10 μm, respectively.

## 3. Results and discussion

**Figure 1.**
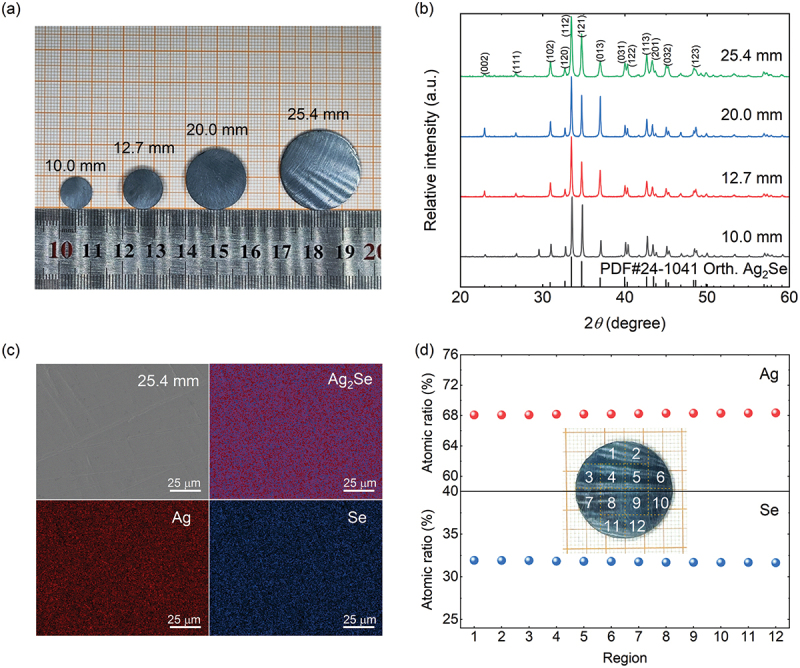
Phase structure and compositional homogeneity of the prepared Ag_2_Se sample. (a) Optic images and (b) XRD patterns of Ag_2_Se samples with different sizes. (c) Surface morphology and elemental distribution, and (d) EPMA results of the *φ*25.4 Ag_2_Se sample.

In order to evaluate the homogeneity of the chemical composition of Ag_2_Se-based materials, different sizes of Ag_2_Se were prepared, and the optical images are shown in Figure 1(a). All samples possess a distinct metallic luster and a high densification with a relative density of ~98% (Table S2, Supplementary Information). The X-ray diffraction patterns presented in Figure 1(b) indicated that the prepared Ag_2_Se materials with different sizes show a single phase with an orthorhombic structure, which is indexed as PDF#24–1041. The full width at half maximum (FWHM) of the Ag_2_Se sample with a diameter of 25.4 mm (*φ*25.4) is 0.2°, further demonstrating good crystallinity. To further evaluate the uniformity of the *φ*25.4 sample, elemental distribution and chemical composition are characterized, and the results are shown in Figure 1(c,d). The elements are distributed homogeneously without obvious elemental segregations in this sample, as shown in Figure 1(c) and Figure S2 (Supplementary Information). The electron probe microanalysis results presented in Figure 1(d) show a highly uniform distribution of chemical composition in the different regions. The measured composition of the sample is Ag_2_Se_0.93_, which is close to the reported values [42].

**Figure 2.**
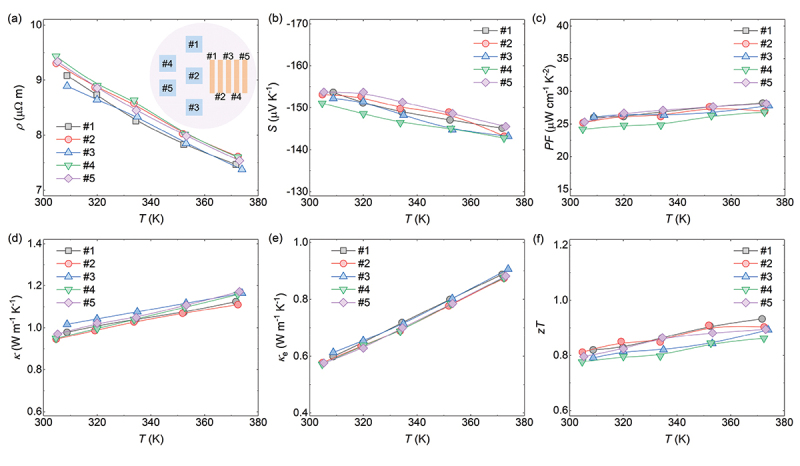
Thermoelectric properties of the *φ*25.4 Ag_2_Se samples. Temperature-dependent (a) electrical resistivity, (b) Seebeck coefficient, (c) power factor, (d) thermal conductivity, (e) the sum of electronic and bipolar thermal conductivity, and (d) *zT* values.

To evaluate the homogeneity of thermoelectric properties, five bar-shaped and five disk-shaped specimens were prepared for measuring electrical properties and thermal conductivity, respectively. As shown in Figure 2(a), the electrical resistivities of Ag_2_Se show a monotonic decrease from ~9.3 μΩ m at 300 K to ~7.5 μΩ m at 380 K. As a narrow bandgap semiconductor [43–45], the bipolar effect induces excitation of minor carriers with the elevation of temperature, leading to an increase in carrier concentration (Figure S3, Supplementary Information) [46,47]. Simultaneously, the Seebeck coefficient exhibited a similar trend with the electrical resistivity as shown in Figure 2(b). The negative Seebeck coefficients of Ag_2_Se indicate n-type semiconductor behavior, which is likely attributed to the intrinsic Se vacancy [48,49]. The Seebeck coefficient of Ag_2_Se decreases with the increase of temperature from −153 μV K^−1^ at 300 K to −145 μV K^−1^ at 380 K, further supporting the onset of bipolar effect. As shown in Figure 2(c), the power factor (*PF* = *S*^2^/*ρ*) of Ag_2_Se shows a slight increase from ~25 μW cm^−1^ K^−2^ at 300 K to ~ 28 μW cm^−1^ K^−2^ at 380 K. Figure 2(d) shows the temperature-dependent thermal conductivity of Ag_2_Se. The total thermal conductivity shows a continuous increase from ~0.95 W m^−1^ K^−1^ at 300 K to ~1.15 W m^−1^ K^−1^ at 380 K. The total thermal conductivity is composed of the lattice thermal conductivity (*κ*_L_), the electronic thermal conductivity (*κ*_e_), and the bipolar thermal conductivity (*κ*_bip_). Due to the bipolar conduction, the enhanced total thermal conductivity should be ascribed to the increased electronic thermal conductivity [50,51] (*κ*_e_ = *LσT*, where *L* is the Lorenz number, calculated by the empirical formula, *L* = 1.5 + exp(|*S*|/115) [52]), and the additional contribution from the bipolar thermal conductivity, as shown in Figure 2(e) and Figure S4, Supplementary Information. Finally, the *zT* values of Ag_2_Se-based materials are around 0.8 at 300 K, and the average *zT* (*zT*_avg_) is ~0.85 at the temperature range from 300 to 380 K, as shown in Figure 2(f). The overall thermoelectric properties of five Ag_2_Se samples are comparable (Figure S5, Supplementary Information). These results demonstrate the homogeneous chemical composition and promising thermoelectric performance of the prepared Ag_2_Se sample.

**Figure 3.**
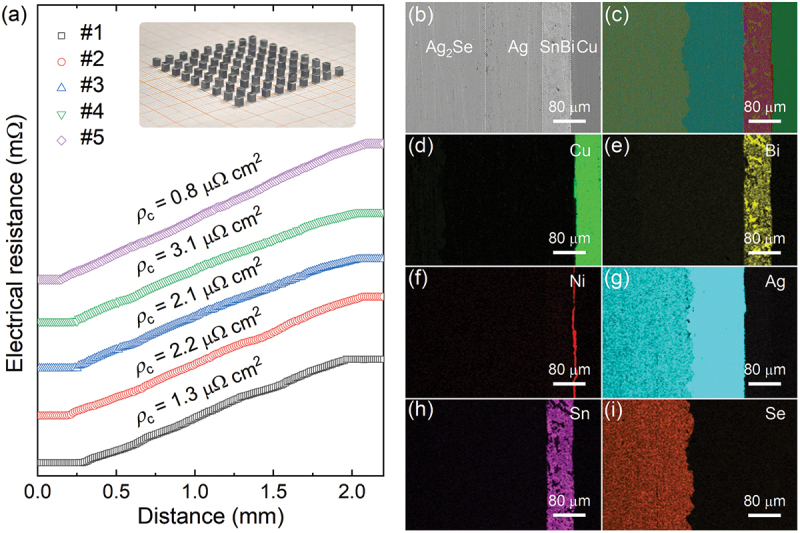
Interfacial properties of Ag_2_Se-based interface. (a) Contact resistivity for five Ag/Ag_2_Se/Ag legs. The inset shows the prepared Ag/Ag_2_Se/Ag legs. (b) The interface of the soldered Ag_2_Se leg joint and (c–i) their corresponding EDS mapping for the interface.

To fabricate the Ag_2_Se-based thermoelectric coolers, the preparation of the Ag/Ag_2_Se/Ag joint was realized by spark plasma sintering, and a residual pressure was maintained during the cooling process to minimize the detrimental effect of phase transition [38]. As depicted in the inset of Figure 3(a), ~90 Ag_2_Se-based legs with dimensions of 1.8 mm × 1.8 mm × 2.6 mm were obtained through cutting a single *φ*25.4 mm Ag/Ag_2_Se/Ag plate [53]. To evaluate the electrical contact resistivity at the Ag/Ag_2_Se/Ag interface, five Ag_2_Se legs were characterized, as shown in Figure 3(a). The contact resistivity of the Ag/Ag_2_Se interface varies from 0.8 to 3.1 μΩ cm^2^, indicating negligible electrical parasitic loss. To be noted that the contact resistivity is lower than that of Ni/Ag_2_Se (*ρ*_c_ of ~ 12 μΩ cm^2^) [37], and comparable with our previous result (*ρ*_c_ of ~2.9 μΩ cm^2^) [38]. The Ag_2_Se-based legs were further bonded with the Cu electrode (deposited with a thin Ni layer) by using the SnBi solder at 458 K. Scanning electron microscopy and energy dispersive spectroscopy were applied to characterize the interface of the soldered joint. Figure 3(b) shows clear and distinct boundaries between the successive layers: Cu electrode/SnBi solder, SnBi solder/Ag contact layer, and Ag contact layer/Ag_2_Se. The contact resistivity of the soldered Ag/Ag_2_Se joint is 1.5 μΩ cm^2^ (Figure S6, Supplementary Information), indicating a good contact property. In addition, the corresponding energy dispersive spectroscopy mapping results (Figure 3(c–h)) reveal the homogeneous elemental distributions without obvious diffusions. Furthermore, the linear EDS scanning across the Ag/Ag_2_Se joint illustrates the distinct interfaces. (Figure S7, Supplementary Information). In addition, there is a narrow diffusion layer between Ag/SnBi boundary, which is beneficial to ensure a good bonding strength (Figure S8, Supplementary Information). The above results confirm that Ag is a good contact layer for Ag_2_Se and the feasibility for the practical fabrication of Ag_2_Se-based thermoelectric coolers.

**Figure 4.**
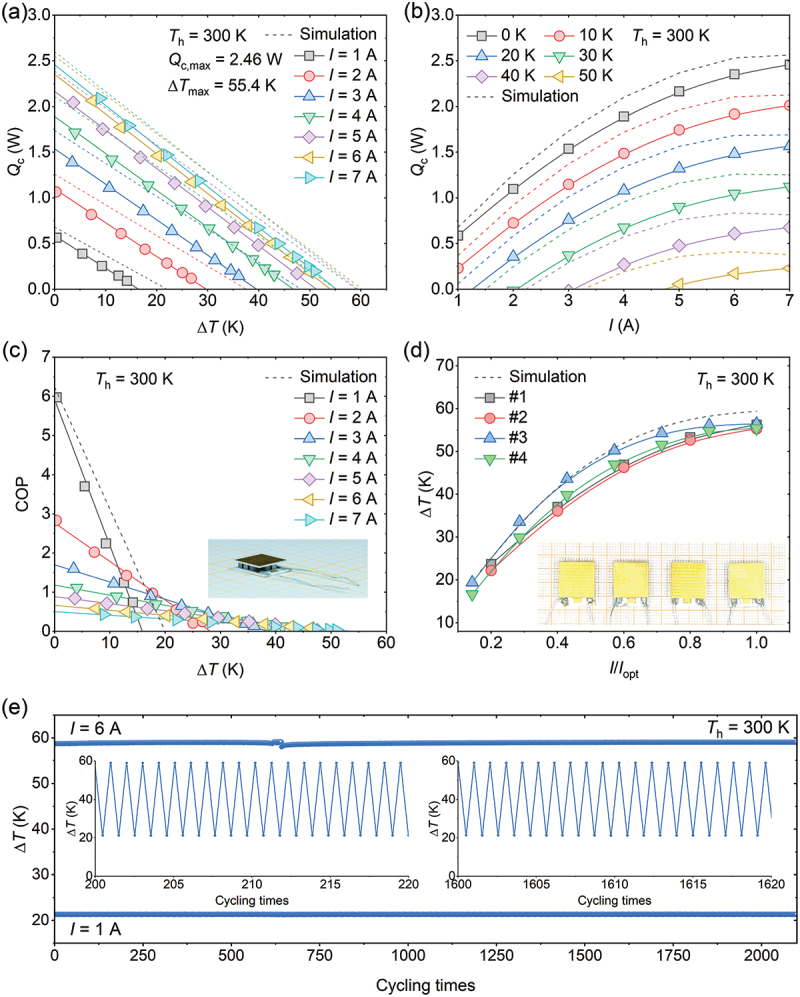
Thermoelectric cooling performance of Ag_2_Se-based device at the hot-side temperature of 300 K. Cooling power as a function of (a) cooling temperature difference at varying electrical currents and (b) electrical current at varying cooling temperature differences. (c) Coefficient of performance as a function of cooling temperature difference. (d) The relationship between cooling temperature difference and electrical currents for four Ag_2_Se-based coolers. (e) Cycling of the Ag_2_Se-based thermoelectric cooler for more than 2000 times between 1 a and 6 A. The insets present the cycling test data.

To further validate the thermoelectric performance of the Ag_2_Se sample, a cooling device based on 7 pairs of n-type Ag_2_Se and p-type (Bi, Sb)_2_Te_3_ was fabricated. The cooling performance of the Ag_2_Se-based device was characterized on a homemade apparatus at the hot-side temperature of 300 K. As shown in Figure 4(a), there is a good linear relationship between the cooling power and cooling temperature difference of the Ag_2_Se-based device. When the applied electrical current was 7 A, the device achieved a maximum cooling power of ~2.46 W, corresponding to a cooling power density of 1.46 W cm^−2^. In addition, at the optimal electrical current, the maximum cooling temperature difference is 55.4 K, which is comparable to the previously reported Ag_2_Se-based coolers [37–39], and lower than Bi_2_Te_3_-based [14,54] and Mg_3_(Sb, Bi)_2_-based devices [30,32,34] (Table S3, Supplementary Information). The relationship between cooling power and electrical current under different cooling temperature differences is presented in Figure 4(b). Correspondingly, the steady-state cooling performance of the Ag_2_Se-based device is simulated, and the impact of thermal radiation is considered (Figure S9, Supplementary Information). The cooling power experiences an increment with the increase in electrical current from 1 to 7 A. In addition, the Ag_2_Se-based device also achieved a maximum cooling power of 2.76 W and a maximum cooling temperature difference of 61.2 K at the hot-side temperature of 325 K (Figure S10-11, Supplementary Information). The cooling coefficient of performance of the Ag_2_Se-based device exhibits a nearly linear relationship with the cooling temperature difference, as shown in Figure 4(c). The inset image shows the side view of the Ag_2_Se-based cooler. The coefficient of performance decreases with the increase in cooling temperature difference. Recently, a criterion for evaluating the measurement uncertainty has been proposed: the coefficient of performance should be 0.5 at the optimum electrical current, under the condition of maximum cooling power and zero cooling temperature difference [55]. Herein, the Ag_2_Se-based device achieved a coefficient of performance of 0.504 at the zero temperature difference and the optimal electrical current of 7 A (Figure S12, Supplementary Information), further supporting the reliability of the thermoelectric cooling performance measurement.

To evaluate the reproducibility of their cooling performance, four Ag_2_Se-based thermoelectric coolers were fabricated and characterized. The cooling temperature differences as a function of electrical current at the hot-side temperatures of 300 K are presented in Figure 4(d). The inset of Figure 4(d) shows the optical image of four Ag_2_Se-based devices. It is clear that the cooling temperature difference increases with the increase in electrical current, and reaches a maximum value of ~ 56 K at the optimal electrical current for four devices. In addition, the operational stability of the Ag_2_Se-based device is also investigated with cycling tests between the electrical currents of 1 A and 6 A at the hot-side temperature of 300 K, and the results are shown in Figure 4(e). It can be noted that the cooling temperature difference at two electrical currents experiences a fluctuation within 2% after more than 2000 cycles. Furthermore, the interface of the Ag_2_Se-based thermoelectric cooler after the cycling is also characterized, as shown in Figure S13 (Supplementary Information). It can be noted that there is a clear boundary between the various interfaces without obvious elemental diffusions, demonstrating a superior stability of the Ag_2_Se-based thermoelectric cooler.

## 4. Conclusion

Herein, the fabrication of the Ag_2_Se-based thermoelectric coolers has been realized. Good compositional homogeneity and excellent thermoelectric performance have been achieved in the *φ*25.4 mm Ag_2_Se sample. Using the spark plasma sintering, the Ag/Ag_2_Se/Ag joint is prepared, and a total amount of ~ 90 Ag/Ag_2_Se/Ag legs can be obtained with a low contact resistivity ranging from 0.8 to 3.1 μΩ cm^2^. Four thermoelectric devices based on the n-type Ag_2_Se and p-type (Bi, Sb)_2_Te_3_ have been fabricated, and they show a maximum cooling temperature difference of ~ 56 K at the hot-side temperature of 300 K. In addition, it also shows excellent operational stability after more than 2000 cycles of electrical currents between 1 A and 6 A. Our results demonstrate that Ag_2_Se-based materials and devices hold great promise for practical applications of electronic refrigeration.

## Supplementary Material

Supplemental Material
