# Supplementary material for: Towards the practical realization of high-performance Ag2Se-based thermoelectric coolers
Source: Sci Technol Adv Mater. 2026 Mar 12;27(1):2641882. doi: 10.1080/14686996.2026.2641882 (PMC13023009; doi:10.1080/14686996.2026.2641882)
Supplement: Supplemental Material [file TSTA_A_2641882_SM7880.docx]

# Supplementary information

# Towards the practical realization of high-performance Ag_2_Se-based thermoelectric coolers

Feng Jiang^a†^, Zhengtao Wang^a†^, Wen Zhong^a^, Yifan Zhou^a^, Zhengyang Zhou^a^, Longzhi Wu^a^, Jiang Chen^a^, Yao Xu^a^, Xiaodong Wang^a,d^, Feng Cao^c^, Qian Zhang^a, b, e*^, and Jun Mao^a, b, e*^

*a School of Materials Science and Engineering, and Institute of Materials Genome & Big Data, Harbin Institute of Technology (Shenzhen), Shenzhen 518055, P.R. China.*

*b State Key Laboratory of Precision Welding & Joining of Materials and Structures, Harbin Institute of Technology, Harbin 150001, P.R. China.*

*c School of Science, Harbin Institute of Technology (Shenzhen), Shenzhen 518055, P.R. China.*

*d Institute of Special Environments Physical Sciences, Harbin Institute of Technology (Shenzhen), Shenzhen 518055, P.R. China.*

*e School of Materials Science and Engineering, Shenzhen Key Laboratory of New Materials Technology, Shenzhen 518055, P.R. China.*

^†^These authors contributed equally to this work.

^*^Corresponding authors, email: zhangqf@hit.edu.cn; [maojun@hit.edu.cn](mailto:maojun@hit.edu.cn);

# Contents

1. **Setting parameters for the finite element simulation of Ag_2_Se-based cooler**
2. **Sample density of Ag_2_Se materials with different sizes**
3. **Specific heat capacity of the Ag_2_Se sample**
4. **Energy dispersive spectroscopy mapping of the Ag_2_Se sample**
5. **Hall carrier concentration and mobility of the Ag_2_Se sample**
6. **The lattice thermal conductivity of the Ag_2_Se sample**
7. **Thermoelectric properties of five Ag_2_Se samples**
8. **Contact resistivity of the soldered Ag/Ag_2_Se joint**
9. **Energy dispersive spectroscopy and linear scanning of the Ag/Ag_2_Se interface**
10. **Linear energy dispersive spectroscopy scanning of the Ag/SnBi interface**
11. **Comparison of thermoelectric cooling performance for Ag_2_Se, Bi_2_Te_3_, and Mg_3_(Sb, Bi)_2_-based devices at the hot-side temperature of 300 K**
12. **The simulated cooling performance of the 7 pairs Ag_2_Se/(Bi, Sb)_2_Te_3_ thermoelectric cooler**
13. **Thermoelectric cooling performance of the Ag_2_Se-based cooler at the hot-side temperature of 325 K**
14. **Coefficient of performance as a function of the electrical current of the Ag_2_Se-based thermoelectric cooler**
15. **Interfacial properties of the Ag_2_Se-based thermoelectric cooler after cycling**

## Setting parameters for the finite element simulation of Ag_2_Se-based cooler

**Table S1. Finite element simulation setting parameters.**

| Parameter | Value |
| --- | --- |
| Cross-sectional area of the Ag_2_Se legs | 1.8 × 1.8 mm^2^ |
| Height of the Ag_2_Se legs | 2.5 mm |
| Cross-sectional area of the (Bi, Sb)_2_Te_3_ legs | 2.0 × 2.0 mm^2^ |
| Height of the (Bi, Sb)_2_Te_3_ legs | 2.5 mm |
| Thickness of copper electrode | 0.065 mm |
| Thickness of Al_2_O_3_ substrate | 0.38 mm |
| The area of the Al_2_O_3_ substrate of the cold side | 13.0 × 13.0 mm^2^ |
| The area of the Al_2_O_3_ substrate of the hot side | 13.0 × 16.0 mm^2^ |
| Thermal conductivity of the substrate | 20 W m^-1^ K^-1^ |
| Thermal conductivity of the thermal grease | 4.8 W m^-1^ K^-1^ |
| Heat load | 0.0-2.5 W |
| Hot-side temperature | 300 K |
| Contact resistivity of Ag_2_Se legs | 5.0 μΩ cm^2^ |
| Contact resistivity of (Bi, Sb)_2_Te_3_ legs | 5.0 μΩ cm^2^ |

## Sample density of Ag_2_Se materials with different sizes

**Table S2.** Sample density of Ag_2_Se materials with different sizes

| Sample diameter  (mm) | Measured density  (g cm^-3^) | Relative density  (%) |
| --- | --- | --- |
| 10.0 | 8.05 | 98.17 |
| 12.7 | 8.07 | 98.41 |
| 20.0 | 8.03 | 97.93 |
| 25.4 | 8.06 | 98.29 |

## Specific heat capacity of the Ag_2_Se sample





**Figure S1**. Specific heat capacity of the Ag_2_Se sample.

## Energy dispersive spectroscopy mapping of the Ag_2_Se sample


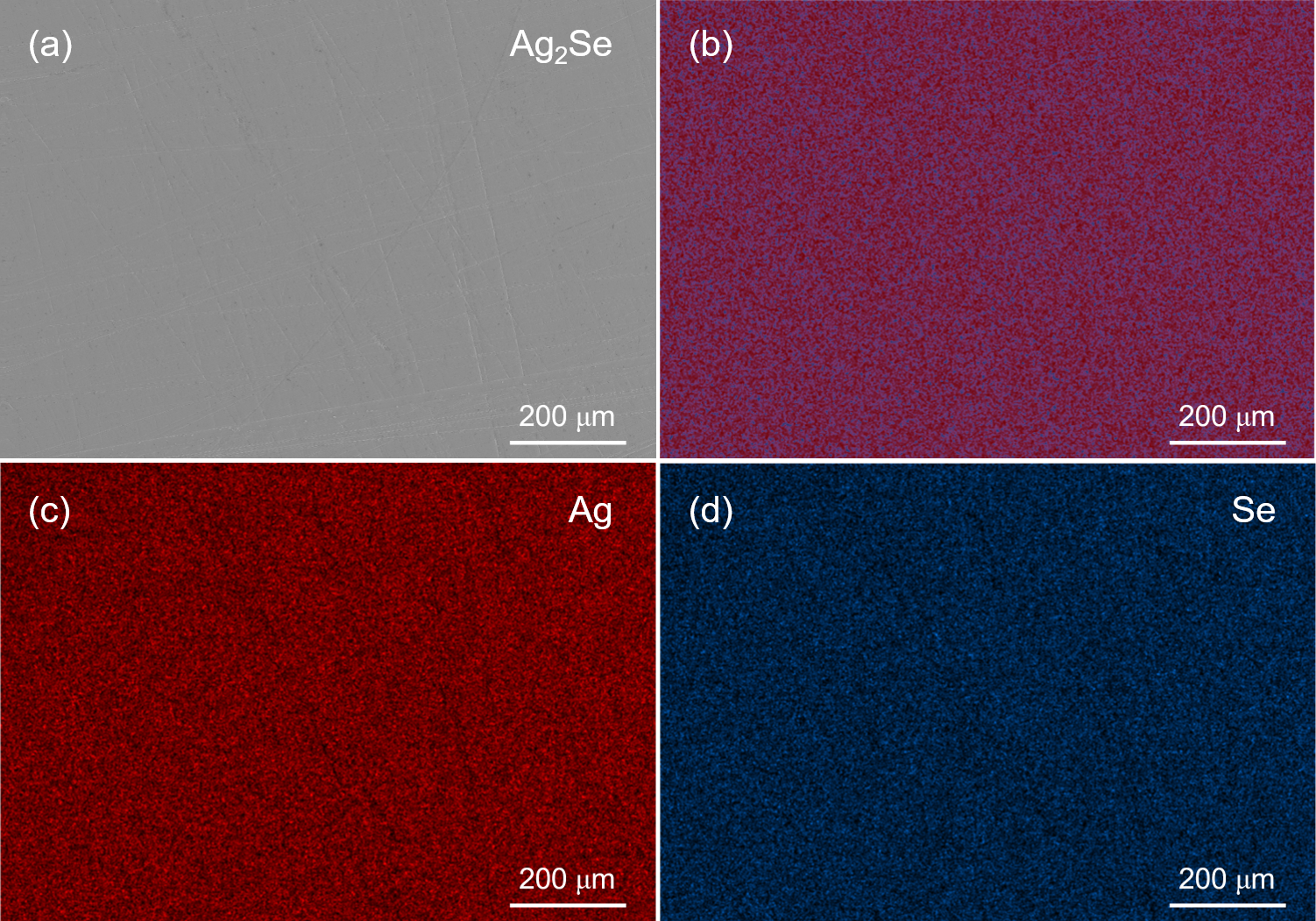


**Figure S2.** (a) Surface morphology, and (b-d) energy dispersive spectroscopy mapping of the Ag_2_Se sample.

## Hall carrier concentration and mobility of the Ag_2_Se sample





**Figure S3.** **Hall carrier concentration and mobility of the Ag_2_Se sample.** (a) Carrier concentration. (b) Carrier mobility.

## The lattice thermal conductivity of the Ag_2_Se sample





**Figure S4.** The lattice thermal conductivity of the Ag_2_Se sample.

## Thermoelectric properties of the Ag_2_Se samples





**Figure S5**. **Thermoelectric properties of the Ag_2_Se samples.** Temperature-dependent (a) electrical resistivity, (b) Seebeck coefficient, (c) thermal conductivity, and (d) *zT* values.

## Contact resistivity of the soldered Ag/Ag_2_Se joint





**Figure S6.** Contact resistivity of the soldered Ag/Ag_2_Se joint.

## Energy dispersive spectroscopy and linear scanning of the Ag/Ag_2_Se interface





**Figure S7.** (a) The Ag/Ag_2_Se interface and (b-d) corresponding EDS mapping for the interface. (e) Linear EDS scanning across the Ag/Ag_2_Se interface.

## Linear energy dispersive spectroscopy scanning of the Ag/SnBi interface





**Figure S8.** Linear EDS scanning across the Ag/SnBi solder interface.

## Comparison of thermoelectric cooling performance for Ag_2_Se, Bi_2_Te_3_, and Mg_3_(Sb, Bi)_2_-based devices at the hot-side temperature of 300 K

**Table S3.** Comparison of thermoelectric cooling performance for Ag_2_Se, Bi_2_Te_3_, and Mg_3_(Sb, Bi)_2_-based devices at the hot-side temperature of 300 K

| n-type leg | p-type leg | Cooling temperature difference (K) | Cooling power density (W cm^-2^) | Reference |
| --- | --- | --- | --- | --- |
| Ag_2_Se | Bi_2_Te_3_ alloys | 55.4 | 1.5 | This work |
| Ag_2_Se | Bi_2_Te_3_ alloys | 57.7 | 1.5 | Jiang *et al.*^[^[^1^](#_ENREF_1)^]^ |
| Ag_2_Se | Bi_2_Te_3_ alloys | 56.0 | 1.4 | Liu *et al.*^[^[^2^](#_ENREF_2)^]^ |
| Ag_2_Se | MgAgSb | 52.0 | 0.8 | Zhao *et al.*^[^[^3^](#_ENREF_3)^]^ |
| Bi_2_Te_3_ | Bi_2_Te_3_ alloys | 70.1 | 1.6 | Sun *et al.*^[^[^4^](#_ENREF_4)^]^ |
| Bi_2_Te_3_ | Bi_2_Te_3_ alloys | 73.9 | 2.2 | Zhao *et al.*^[^[^5^](#_ENREF_5)^]^ |
| Mg_3_(Sb, Bi)_2_ | Bi_2_Te_3_ alloys | 69.0 | 1.3 | Ma *et al.*^[^[^6^](#_ENREF_6)^]^ |
| Mg_3_(Sb, Bi)_2_ | MgAgSb | 52.0 | 0.8 | Xie *et al.*^[^[^7^](#_ENREF_7)^]^ |
| Mg_3_(Sb, Bi)_2_ | MgAgSb | 61.0 | - | Zhang *et al.*^[^[^8^](#_ENREF_8)^]^ |

## The simulated cooling performance of the 7 pairs Ag_2_Se/(Bi, Sb)_2_Te_3_ thermoelectric cooler





**Figure S9.** **The simulated cooling performance of the 7 pairs Ag_2_Se/(Bi, Sb)_2_Te_3_ thermoelectric cooler.** Cooling power as a function of (a) the temperature difference at different electrical currents and (b) electrical current at different temperature differences. (c) The coefficient of performance as a function of the temperature difference at different electrical currents. (d) The maximum cooling temperature difference at different electrical currents.

## Thermoelectric cooling performance of the Ag_2_Se-based cooler at the hot-side temperature of 325 K





**Figure S10.** Cooling power as a function of (a) cooling temperature difference at different electrical currents, and (b) electrical current at different cooling temperature differences.





**Figure S11.** The relationship between cooling temperature difference and electrical current at the hot-side temperature of 300 and 325 K.

## Coefficient of performance as a function of the electrical current of the Ag_2_Se-based thermoelectric cooler





**Figure S12**. Coefficient of performance as a function of the electrical current of the Ag_2_Se-based thermoelectric cooler at the hot-side temperature of 300 K.

## Interfacial properties of the Ag_2_Se-based thermoelectric cooler after cycling


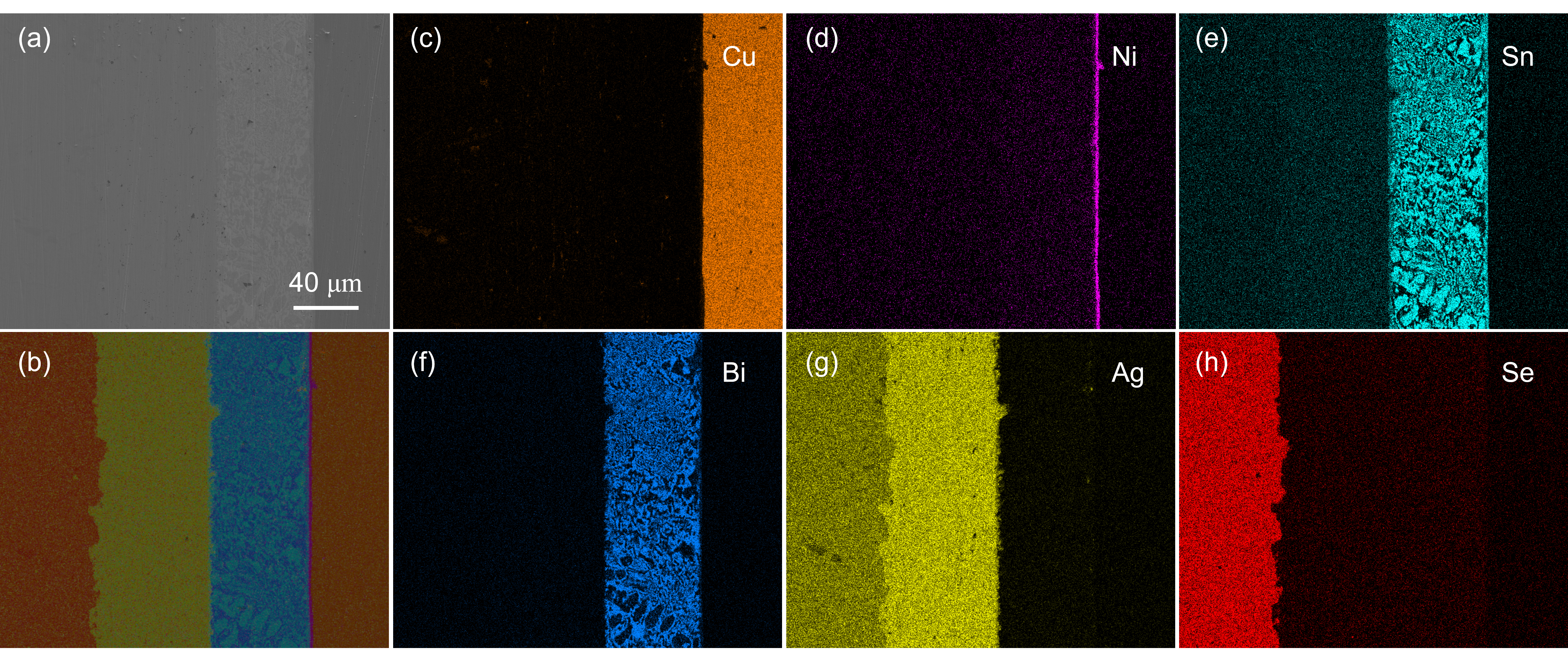


**Figure S13.** (a) Interfacial morphology, and (b-h) energy dispersive spectroscopy mapping of the interface of the Ag_2_Se-based thermoelectric cooler.

References:

[1] Jiang F, Lin CH, Cheng JX, et al. Prefer-oriented Ag_2_Se crystal for high-performance thermoelectric cooling. Adv Funct Mater. (**2024**);35(6):2415000. doi: 10.1002/adfm.202415000

[2] Liu M, Zhang XY, Zhang SX, et al. Ag_2_Se as a tougher alternative to n-type Bi_2_Te_3_ thermoelectrics. Nat Commun. (**2024**);15(1):6580. doi: 10.1038/s41467-024-50898-6

[3] Zhao SY, Shi XL, Zhou Q, et al. Substitution energy-guided screening of diffusion barrier materials for Ag_2_Se-based thermoelectric coolers. Nano Res. (**2025**);18(10):94907903. doi: 10.26599/NR.2025.94907903

[4] Sun YX, Wu H, Dong XY, et al. High performance BiSbTe alloy for superior thermoelectric cooling. Adv Funct Mater. (**2023**);33(28):2301423. doi: 10.1002/adfm.202301423

[5] Zhang Y, Xu G, Nozariasbmarz A, et al. Thermoelectric cooling performance enhancement in BiSeTe alloy by microstructure modulation via hot extrusion. Small Sci. (**2023**);4(2):2300245. doi: 10.1002/smsc.202300245

[6] Ma XJ, Lin CH, Yang HY, et al. Elevating thermoelectric performance in the sub-ambient temperature range for electronic refrigeration. Innovation. (**2025**);6(5):100864. doi: 10.1016/j.xinn.2025.100864

[7] Xie LJ, Yang JW, Liu ZY, et al. Highly efficient thermoelectric cooling performance of ultrafine-grained and nanoporous materials. Mater Today. (**2023**);65(4):5-13. doi: 10.1016/j.mattod.2023.03.021

[8] Zhang XF, Zhu HT, Dong XJ, et al. High-performance MgAgSb/Mg_3_(Sb,Bi)_2_-based thermoelectrics with *η* = 12% at T ≤ 583K. Joule. (**2024**);8(12):3324-3335. doi: 10.1016/j.joule.2024.08.013
